# Baseline angiopoietin‐2 and FGF19 levels predict treatment response in patients receiving multikinase inhibitors for hepatocellular carcinoma

**DOI:** 10.1002/jgh3.12339

**Published:** 2020-04-11

**Authors:** Taku Shigesawa, Goki Suda, Megumi Kimura, Tomoe Shimazaki, Osamu Maehara, Ren Yamada, Takashi Kitagataya, Kazuharu Suzuki, Akihisa Nakamura, Masatsugu Ohara, Machiko Umemura, Naoki Kawagishi, Masato Nakai, Takuya Sho, Mitsuteru Natsuizaka, Kenichi Morikawa, Koji Ogawa, Naoya Sakamoto

**Affiliations:** ^1^ Department of Gastroenterology and Department of Hepatology, Graduate School of Medicine Hokkaido University Sapporo Japan

**Keywords:** angiopoietin‐2, fibroblast growth factor 19, hepatocellular carcinoma, sorafenib, treatment outcome

## Abstract

**Background:**

Sorafenib and lenvatinib are first‐line systemic therapies for unresectable hepatocellular carcinoma (HCC). However, the criteria for their selection remain unclear.

**Methods:**

We identified patients with unresectable HCC who were treated with sorafenib or lenvatinib between August 2009 and January 2019 at the Hokkaido University Hospital. Patients who continued treatment for >2 months, underwent evaluation by computed tomography every 2–3 months, and had complete clinical data were included. Responders were patients with objective response (OR) for lenvatinib and patients with stable disease (SD) exceeding 6 months (long‐SD) or OR for sorafenib. The predictive factors for treatment response, including fibroblast growth factor (FGF)19 and 21, angiopoietin 2 (ANG2), hepatocyte growth factor, and vascular endothelial growth factor, were evaluated.

**Results:**

Overall, 27 and 29 patients treated with lenvatinib and sorafenib, respectively, were included. The responders for lenvatinib and sorafenib were 63% (17/27) and 38% (11/29), respectively. No significant predictive factors for treatment response were identified in patients treated with sorafenib. However, baseline serum FGF19 and ANG2 levels were significantly associated with treatment response to lenvatinib. All (9/9) patients with low baseline ANG2 and FGF19 levels who received lenvatinib achieved OR. Conversely, the OR was low (13%; 1/9) in patients with high baseline ANG2 and FGF19 levels. Responder rate was 40% (2/5) in patients with high baseline ANG2 and FGF19 levels who received sorafenib.

**Conclusion:**

This study is, to our knowledge, the first to demonstrate that baseline ANG2 and FGF19 levels may aid in selecting optimal systemic therapy for patients with unresectable HCC.

## Graphical abstract

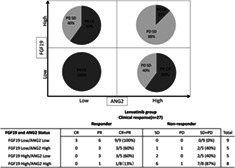

## Introduction

Hepatocellular carcinoma (HCC) is the sixth most common cancer and the third most common cause of cancer‐related deaths worldwide.^1^ Owing to the limited therapeutic options, the prognosis of patients with unresectable HCC remains poor. Until recently, the multikinase inhibitor sorafenib was the only approved systemic treatment for these patients.^2^

As subsequent clinical trials on novel systemic therapy failed to show significant efficacy or noninferiority to sorafenib,^3^, ^4^, ^5^ it has long remained the only approved systemic therapy for patients with unresectable HCC. However, recently, the multikinase inhibitor regorafenib was approved as second‐line systemic therapy for patients with advanced HCC.^6^ REFLECT, a phase 3 clinical trial of the multikinase inhibitor lenvatinib, demonstrated noninferior overall survival (OS) compared to sorafenib in unresectable HCC.^7^ Therefore, either multikinase inhibitor may be selected as first‐line therapy; however, criteria for their selection based on patient and tumor characteristics are unavailable. Reports suggest that the objective response (OR), evaluated by modified Response Evaluation Criteria in Solid Tumors (mRECIST), is an independent prognostic factor for OS with systemic therapy in these cases.^8^, ^9^, ^10^ In addition, in patients receiving sorafenib for unresectable HCC, the impact of long‐term stable disease (long‐SD) on OS is similar to that of complete response (CR) and partial response (PR).^8^

Various studies have analyzed the predictive factors of the treatment response to sorafenib in unresectable HCC. However, till date, validated predictive factors have not been established.^11^ No predictive biomarkers for treatment response to sorafenib were identified in the largest biomarker study cohort, the SHARP trial.^12^ However, considering the difference in kinase affinity profiles between sorafenib, which targets the Raf/MEK/ERK pathway, RTKs, VEGFR1, VEGFR2, VEGFR3, and PDGFR‐β, and lenvatinib, which targets VEGFR1–3, FGFR1–4, PDGFR‐α, c‐Kit, and RET, it is possible that certain biomarkers may be available to determine multikinase inhibitor suitability depending on patient profiles.

In this study, we aimed to evaluate the predictive factors of the treatment response to lenvatinib in patients with unresectable HCC. We also intended to propose criteria for the selection of multikinase inhibitors based on baseline patient characteristics.

## Materials and methods

### *Patients and study design*

For this retrospective study, we identified patients with unresectable HCC who were treated with sorafenib or lenvatinib between August 2009 and January 2019 at the Hokkaido University Hospital. Patients who met the diagnostic criteria for advanced HCC according to the American Association for the Study of Liver Diseases guidelines^13^ were treated for more than 2 months after treatment initiation, underwent response evaluation using dynamic computed tomography (CT) at baseline and at 2–3‐month intervals, and had adequate clinical data and baseline preserved serum samples for evaluation of serum biomarkers were included. Patients were excluded if they received sorafenib or lenvatinib in conjunction with other treatment, including transarterial chemoembolization (TACE), hepatic arterial infusion chemotherapy (HAIC), and other antitumor agents; were followed up for less than 2 months; had decompensated liver cirrhosis; had insufficient clinical data or no baseline preserved serum samples; and were not evaluated for treatment response using dynamic CT.

We collected data on gender, age, etiology of HCC, blood cell counts, laboratory data, tumor makers (alpha‐fetoprotein [AFP] and des‐gamma‐carboxyprothrombin), Barcelona Clinic Liver Cancer (BCLC) stage, presence of extrahepatic metastases, and Child‐Pugh score at baseline. In addition, we evaluated the baseline levels of the candidate serum biomarkers, including fibroblast growth factor (FGF) 19 and 21, angiopoietin 2 (ANG2), hepatocellular growth factor (HGF), and vascular endothelial cell growth factor (VEGF). Serum FGF19, FGF21, ANG2, HGF, and VEGF levels were estimated using commercial ELISA according to the manufacturer's protocols (FGF19: R&D Systems, Minneapolis, MN, USA; FGF21: Merck Millipore, Darmstadt, Germany; ANG2: R&D Systems, Minneapolis, MN, USA; HGF: R&D Systems, Minneapolis, MN, USA; and VEGF: R&D Systems, Minneapolis, MN, USA).

Patients were routinely assessed using laboratory tests and physical findings every 2 weeks and enhanced CT every 2–3 months after treatment initiation.

### *Treatment protocol*

Sorafenib was administered orally at a dose of 800 mg once daily. Lenvatinib was also administered orally; the dose was determined based on the body weight, with once‐daily doses of 8 and 12 mg for those who weighed <60 and ≥ 60 kg, respectively.

Sorafenib and lenvatinib were discontinued in cases of unacceptable adverse events (AEs) or disease progression. Doses were adjusted based on the occurrence of AEs and tolerability as evaluated by the attending physician.

### *Evaluation of treatment response to either agent*

The treatment responses were evaluated every 2–3 months after treatment initiation by the attending physician using dynamic CT based on the mRECIST criteria.^14^ Based on the criteria, CR and PR were defined as disappearance of any intratumoral arterial enhancement in all target lesions and at least a 30% decrease in the sum of diameters of viable (enhancement in the arterial phase) target lesions, respectively, taking as reference the baseline sum of the diameters of target lesions. Progressive disease (PD) was defined as an increase of at least 20% in the sum of the diameters of viable (enhancing) target lesions, taking as reference the smallest sum of the diameters of viable (enhancing) target lesions recorded since treatment started. Tumors not meeting the criteria for CR, PR, and PD were considered to be SD.

According to recent reports, OR based on mRECIST is an independent prognostic factor for OS in patients receiving systemic therapy for unresectable HCC.^9^ Therefore, in the lenvatinib group, those with OR (CR and PR) were defined as responders. As OR rates with sorafenib are lower than those with lenvatinib,^7^ and the impact of long‐SD on OS in those receiving sorafenib is similar to that of OR with sorafenib,^8^ patients with SD for >6 months (long‐SD) and PR/CR with sorafenib were considered responders; other patients were defined as nonresponders (nonresponder).

### *Statistical analysis*

Continuous variables were analyzed using the paired Mann–Whitney *U*‐test, while categorical variables were analyzed using the chi‐square and Fisher's exact tests. *P* < 0.05 was considered statistically significant. The cut‐off point was based on the receiver operating characteristics (ROC) curve by maximizing the Youden index. The relationship between two variables was analyzed by Spearman's rank correlation. Statistical analyses were performed using the Prism 7.03 (GraphPad Software, Inc., La Jolla, CA, USA) software packages.

### *Informed consent in studies with human subjects*

This study conformed to the ethical guidelines of the Declaration of Helsinki and was approved by the ethics committees of the Hokkaido University Hospital (approval number: 017‐0521). All participating patients provided written informed consent to participate in the study and were provided the option to decline participation.

## Results

### *Enrolled patients and baseline characteristics*

Treatment with sorafenib was initiated in 80 patients with unresectable HCC at the Hokkaido University Hospital between August 2009 and September 2017. Among them, 51 were excluded from analysis as 26, 21, and 4 patients who were treated with combined TACE, HAIC, or anticancer agents discontinued therapy within 2 months due to AEs and were lost to follow up. Finally, 29 patients with unresectable HCC who had received sorafenib were included in this study.

Treatment with lenvatinib was initiated in 34 patients with unresectable HCC. Among them, seven patients were excluded from analysis as six and one patients discontinued therapy within 2 months due to AEs or were treated with other anticancer agents, respectively. Finally, 27 patients with unresectable HCC who had been treated with lenvatinib were included in this study (Fig. S1).

The patients' baseline characteristics are shown in Table S1. As evident from the table, except for median age (69 and 63 years in lenvatinib and sorafenib groups, respectively; *P* < 0.01), presence of extrahepatic metastases (25% and 55% in lenvatinib and sorafenib groups, respectively; *P* < 0.05), and median platelet counts (16.0 and 10.3 in lenvatinib and sorafenib groups, respectively; *P* < 0.05), the baseline characteristics were similar in both groups.

### *Treatment response*

The treatment response in the lenvatinib group was evaluated based on the best overall response.

As shown in Table 1, among 27 patients, 3 (11%), 13 (48%), 9 (33%), and 2 (7%) experienced CR, PR, SD, and PD, respectively. Overall, the OR rate in the lenvatinib group (i.e., the total proportion of patients with CR and PR) was 59% (16/27).

**Table 1:** Treatment response

Sorafenib group; Clinical response (*n* = 29)						
Response				Nonresponse		
CR	PR	Long SD	CR + PR + Long SD	Short SD	PD	Short SD + PD
0/29 (0%)	2/29 (7%)	9/29 (31%)	11/29 (38%)	10/29 (34%)	8/29 (28%)	18/29 (62%)

Lenvatinib group; Clinical response (*n* = 27)					
Response			Non‐response		
CR	PR	CR + PR	SD	PD	SD + PD
3/27 (11%)	13/27 (48%)	16/27 (59%)	9/27 (33%)	2/27 (7%)	11/27 (41%)

CR, complete response, PD, progressive disease; PR, partial response, SD, stable disease.

In the sorafenib group, treatment response was categorized as responder (SD exceeding 6 months or CR/PR) and nonresponder (SD less than 6 months or PD).

As shown in the Table 1, among 29 patients, 0 (0%), 2 (7%), 19 (67%), and 8 (28%) showed CR, PR, SD, and PD, respectively. In patients with SD (n = 19), nine experienced SD exceeding 6 months. Overall, 11 (38%) and 18 (62%) patients were responders and nonresponders, respectively.

### *Relationship between baseline serum growth factor and treatment response*

**Figure 1:**
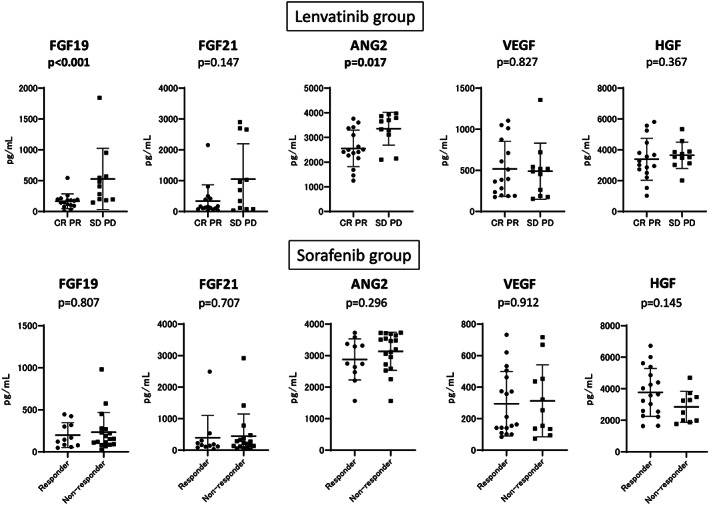
Comparison of baseline serum growth factors between responders and nonresponders in the sorafenib and lenvatinib groups. Baseline FGF19, FGF21, ANG2, VEGF, and HGF were measured in both treatment groups. We compared the mean values between the responders and nonresponders (CR/PR *vs* SD/PD and responder *vs* nonresponder in the lenvatinib and sorafenib groups, respectively). *P* < 0.05 was considered statistically significant.

We analyzed the baseline levels of the growth factors (FGF19, FGF21, HGF, ANG2, and VEGF) in those who received lenvatinib or sorafenib. We subsequently analyzed the relationship between baseline growth factor levels and treatment response in both groups. As shown in Figure 1, in patients who received sorafenib, all the baseline levels of the candidate biomarkers were similar between the responders and nonresponders. In patients treated with lenvatinib, the baseline FGF19 and ANG2 levels were significantly higher in those with an OR (CR and PR) than in those with non‐OR (SD and PD).

### *Factors associated with treatment response in patients from either group*

Among baseline clinical factors, laboratory data, tumor markers, and growth factor levels, we identified the factors associated with treatment response. As shown in Table 2, in patients treated with sorafenib, none of the clinical factors, growth factors, and tumor marker levels were significantly associated with treatment response. Conversely, as shown in Table 3, baseline serum ANG2 and FGF19 levels alone were significantly associated with treatment response in the lenvatinib group.

**Table 2:** Comparison between responders and nonresponders to sorafenib

	Responder (*n* = 11)	Nonresponder (*n* = 18)	*P*‐value
Baseline characteristics			
Age (year)	68 (47–77)	61 (38–89)	0.081
Gender (male/female)	10‐Jan	16‐Feb	>0.999
Etiology—no. (%)			0.788
HBV	6 (55%)	11 (61%)	
HCV	3 (27%)	3 (17%)	
NBNC	2 (18%)	4 (22%)	
Vascular invasion‐no. (%)	3 (27%)	8 (44%)	0.448
Extrahepatic extension‐no. (%)	4 (36%)	12 (67%)	0.142
BCLC stage‐no. (%)			0.667
B	4 (36%)	5 (28%)	
C	7 (64%)	13 (72%)	
Child‐Pugh class‐no.(%)			0.362
A	10 (91%)	13 (72%)	
B	1 (9%)	5 (28%)	
Biochemical analysis			
Albumin (g/dL)	3.9 (2.8–4.5)	3.7 (2.8–4.5)	0.367
Total bilirubin (mg/dL)	0.9 (0.5–1.7)	0.9 (1.0–2.1)	0.648
Prothrombin time (%)	98.0 (65–122)	76.5 (44–124)	0.162
Platelet (*10^4^/μL)	10.7 (5.9–31.6)	9.8 (4.5–27.5)	0.816
Alpha‐fetoprotein (ng/mL)	19.0 (3–1979)	1195.5 (2–221 328)	0.089
AFP (L3%)	25.1 (0.5–79.6)	12.8 (0–64.9)	0.097
PIVKA‐II (mAU/mL)	312 (20–5361)	2176 (60–198 425)	0.063
FGF19‐pg/mL	76.5 (26.9–434.1)	153.0(27.6–980.5)	0.807
FGF21 (pg/mL)	161.1(30.9–2491.4)	215.4(15.9–2918.9)	0.707
ANG2 (pg/mL)	2747 (1566–3724)	3333 (1563–3728)	0.296
VEGF (pg/mL)	253.7 (73.6–717.2)	187.7 (84.0–732.2)	0.912
HGF (pg/mL)	2869.5 (1760.8–4696.7)	3612.8 (1630.1–6723.5)	0.145

AFP, alpha‐fetoprotein; ANG2, angiopoietin 2; BCLC, Barcelona clinic liver cancer; FGF, fibroblast growth factor; HBV, hepatitis B virus; HCV, hepatitis C virus; HGF, hepatocyte growth factor; NBNC, non‐B non‐C; SD, stable disease, VEGF, vascular endothelial growth factor.

**Table 3:** Comparison between responders and nonresponders to lenvatinib

	PR CR (*n* = 16)	PD SD (*n* = 11)	*P*‐Value
Baseline characteristics			
Age (years)	67 (54–79)	71 (56–83)	0.075
Gender (male/female)	16/0	09‐Feb	0.156
Etiology no. (%)			0.116
HBV	8 (50%)	2 (18%)	
HCV	4 (25%)	2 (18%)	
NBNC	4 (25%)	7 (64%)	
Vascular invasion‐no. (%)	4 (25%)	3 (27%)	>0.999
Extrahepatic extension‐no. (%)	4 (25%)	2 (18%)	>0.999
BCLC stage‐no. (%)			0.124
B	8 (50%)	2 (18%)	
C	8 (50%)	9 (82%)	
Child‐Pugh class‐no. (%)			0.661
A	13 (81%)	8 (73%)	
B	3 (19%)	3 (27%)	
Biochemical analysis			
Albumin (g/dL)	3.8 (2.8–4.6)	3.3 (3–3.8)	0.297
Total bilirubin (mg/dL)	0.7 (0.3–1.9)	0.7 (0.4–3.1)	0.516
Prothrombin time (%)	90.3 (59.2–117.1)	88.4 (46.6–107.5)	0.761
Platelet (*10^4^/μL)	15.0 (6.5–34.6)	19.1 (4.4–51.7)	0.488
Alpha‐fetoprotein (ng/mL)	9.3 (1.6–94 134.4)	97.5 (5.5–449 909)	0.077
AFP (L3%)	10.5 (0.5–99.5)	40.9 (0.5–81)	0.598
PIVKA‐II (mAU/mL)	1807 (13–17 526)	696 (24–195 319)	0.798
FGF19 (pg/mL)	160.5 (5.1–543.5)	408.5 (146.5–1843.4)	**<0.001**
FGF21 (pg/mL)	141.4 (21.9–2156)	690.2 (30.8–2897.8)	0.147
ANG2 (pg/mL)	2501 (1463.1–3761.3)	3651 (2103.7–3972.2)	**0.017**
VEGF (pg/mL)	390.6 (176.8–1104.5)	488.1 (154.1–1356.7)	0.827
HGF (pg/mL)	3150.3 (1023.8–5809.1)	3552.8 (2017.6–5344.7)	0.367

A *p*‐value less than 0.05 is statistically significant. AFP, alpha‐fetoprotein; ANG2, angiopoietin 2; BCLC, Barcelona clinic liver cancer; CR, complete response; FGF, fibroblast growth factor; HBV, hepatitis B virus; HCV. hepatitis C virus; HGF, hepatocyte growth factor; NBNC, non‐B non‐C; PD, progressive disease; PIVKA‐II, protein induced by vitamin K absence or antagonist‐II; PR, partial response; SD, stable disease; VEGF, vascular endothelial growth factor.

### *Classification of treatment response based on combined baseline serum* *ANG2* *and* *FGF19* *levels in patients treated with lenvatinib*

**Figure 2:**
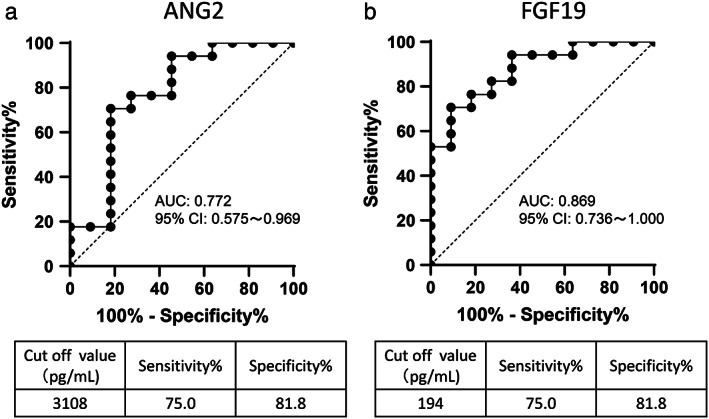
Cut‐off value of baseline serum ANG2 and FGF19 levels for predicting response to lenvatinib. (a) Receiver operating characteristics (ROC) curve analysis for baseline ANG2 levels in patients treated with lenvatinib. The cut‐off baseline ANG2 level associated with response to lenvatinib was set at 3108 pg./mL (ROC‐AUC = 0.772; sensitivity: 75.0%; specificity: 81.8%). (b) ROC curve analysis for baseline FGF19 levels in patients treated with lenvatinib. The cut‐off baseline FGF19 level associated with response was set at 194 pg./mL (ROC‐AUC = 0.869; sensitivity: 75.0%; specificity: 81.8%).

As the baseline FGF19 and ANG2 levels were significantly associated with treatment response in patients treated with lenvatinib, we conducted receiver operating characteristics (ROC) analysis to determine the optimal cut‐off value of baseline ANG2 and FGF19 that were associated with treatment response. As shown in Figure 2, the baseline cut‐off values of ANG2 and FGF19 for predicting treatment response were 3108 pg./mL (sensitivity: 0.75, specificity: 0.818, receiver operating characteristic curve (ROC)‐area under the curve (AUC): 0.772, *P* < 0.001) and 194 pg./mL (sensitivity: 0.75, specificity 0.818, ROC‐AUC: 0.869, *P* < 0.001), respectively.

According to these cut‐off values, the OR rate of lenvatinib was 86% (12/14) in patients with low ANG2 levels; conversely, in those with high levels, the OR rate was 31% (4/13) (Fig. S2). In patients with low FGF19 levels, the OR rate of lenvatinib was 86% (12/14), whereas in those with high levels, the corresponding rate was 31% (4/13) (Fig. S2).

**Figure 3:**
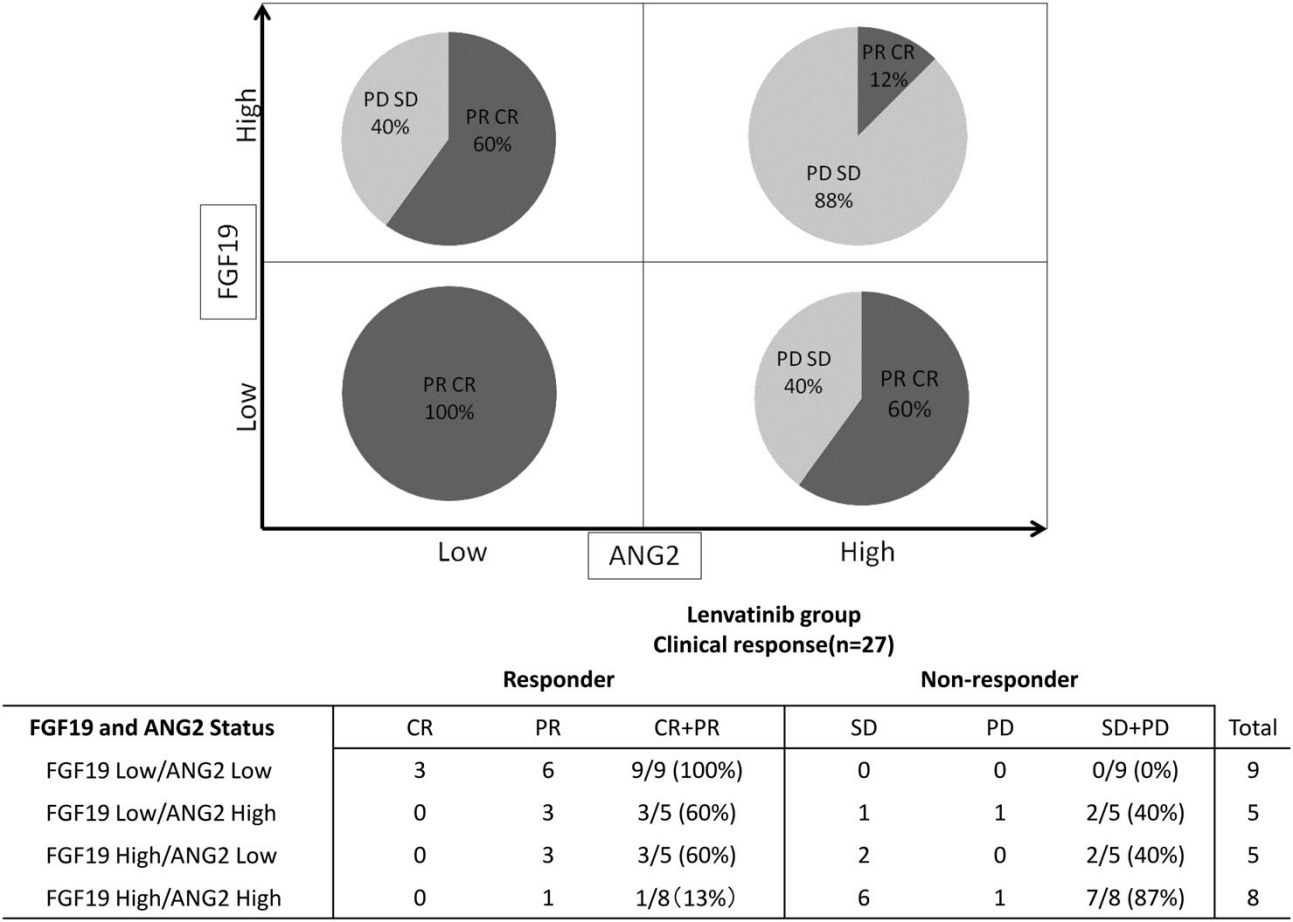
Treatment response to lenvatinib based on baseline ANG2 and FGF19 levels. In patients with low baseline FGF19 and ANG2 levels, the objective response rate (ORR) was 100% (9/9). Conversely, in those with high levels of both markers, the ORR to lenvatinib was 13% (1/8).

We also analyzed the treatment response to lenvatinib based on combined baseline ANG2 and FGF19 levels. As shown in Figure 3, in patients with low baseline FGF19 and ANG2 levels, the OR rate was 100% (9/9); in those with high levels, the rate was 13% (1/8).

We then analyzed the treatment response to sorafenib based on these cut‐off values of baseline FGF19 and ANG2. As shown in Figure S3, treatment responses were similar irrespective of baseline ANG2 and FGF19 levels. However, notably, in patients with high baseline ANG2 and FGF19 levels, the responder rate was 40% (2/5).

### *Characteristics of patients with low and high baseline* *ANG2* *and* *FGF19* *levels*

We finally analyzed the characteristics of those with high baseline ANG2 and FGF19 levels. As shown in Table S2, the baseline albumin and HGF levels were significantly lower and higher than that of the other patients.

## Discussion

The results of the REFLECT trial and clinical data have widened the therapeutic options for patients with unresectable HCC.^7^, ^15^ Currently, patients with unresectable HCC may be treated with either sorafenib or lenvatinib in the first line. However, to date, criteria for their selection based on patients' characteristics have not been established. The response to multikinase inhibitors determines the prognosis of patients with unresectable HCC^8^, ^9^, ^10^; optimizing drug selection is therefore of particular importance. We speculated that, because these two multikinase inhibitors have different kinase affinity profiles, it is possible that certain factors may be identified to aid in individualizing treatment. Therefore, in the present study, we analyzed the predictive factors of treatment response to sorafenib and lenvatinib, focusing on the different kinase affinity profiles between sorafenib and lenvatinib, especially FGFs. Thus, we measured serum FGF2, 19, and 21. However, we could not detect serum FGF2 sufficiently (data not shown); thus, we evaluated serum FGF19 and 21. In addition, previous reports indicated that ANG2,^16^ HGF,^16^, ^17^ and VEGF^17^ could be predictive factor candidates of response to multikinase inhibitors. Therefore, in the present study, we analyzed those serum growth factors.

Similar to the findings of a previous study,^12^ we did not identify significant predictive factors in patients treated with sorafenib. Conversely, as shown in Figure 1, the baseline serum FGF19 and ANG2 levels predicted treatment response to lenvatinib. Additional analysis revealed that, by using the cut‐off values of ANG2 and FGF19, the treatment response could be clearly classified (Figs S2 and S3). In patients with low baseline levels of both ANG2 and FGF19, all (9/9) experienced OR with lenvatinib. On the contrary, in patients with high baseline levels of both ANG2 and FGF19, only 13% (1/8) experienced OR. Notably, the baseline ANG2 and FGF19 levels did not affect treatment response to sorafenib. In patients with high baseline levels of both ANG2 and FGF19, those responding to sorafenib was not low; 40% patients were responders. Therefore, combined baseline ANG2 and FGF19 levels may be a useful biomarker for selecting multikinase inhibitors in patients with unresectable HCC.

Tie2‐mediated signaling is reported to be associated with vessel stabilizing. ANG‐2 is a context‐dependent antagonist of Tie2‐mediated signaling^18^, ^19^; therefore, increased serum levels of ANG2 may cause vascular leaks and metastases. Elevated serum ANG‐2 levels are sometimes observed in patients with HCC.^20^ Increased ANG2 confers poor prognosis in patients with unresectable HCC^12^; however, its role as a predictive biomarker for sorafenib therapy remains controversial.^11^ In addition, the impact of increased ANG2 levels on treatment response to lenvatinib has not been elucidated. Therefore, this study was the first to demonstrate the relationship between ANG2 levels and treatment response in patients receiving lenvatinib for unresectable HCC.

In view of the differences in kinase affinity profiles between sorafenib and lenvatinib, we speculated that FGF signaling may influence the differences in antitumor activity between the two drugs. Therefore, in the present study, we evaluated FGF19 and 21 as candidate biomarkers. FGF signaling is involved in the pathogenesis of HCC. Elevated FGF2 has been observed in patients with advanced liver fibrosis^21^ and has been reported to be involved in the development and progression of HCC.^21^, ^22^, ^23^ In addition to other FGFs, FGF19 and 21 function as endocrine hormones^22^; therefore, they may be suitable serum biomarker candidates. Numerous reports have suggested the central role of FGF19‐mediated signaling via FGFR4 in the pathogenesis of HCC.^22^, ^24^, ^25^, ^26^, ^27^, ^28^ FGF19‐/FGFR4‐mediated signaling is reported to strongly promote HCC proliferation; serum FGF19 is therefore believed to be an important candidate biomarker for treatment response to lenvatinib. As shown in Figure 1, in patients treated with lenvatinib, FGF19 is one of the predictive factors for treatment response. However, as the precise mechanism for the association between increased FGF19 levels and resistance to lenvatinib has not been elucidated, we offer some hypotheses. Lenvatinib may inhibit FGF19‐mediated signaling by blocking FGFR phosphorylation; extremely high FGF19 levels may overcome the effect. In patients with high FGF19 and low ANG2 (Fig. 3), the OR rate was not low (60%, 3/5); conversely, in those with high FGF19 and ANG2 levels, the OR rate was considerably lower (13% 1/8). Therefore, high levels of both FGF19 and ANG2 may strongly suggest nonresponse to lenvatinib. As shown in Table S2, in patients with high levels of both FGF19 and ANG2, the levels of HGF were significantly higher than that of the other enrolled patients, indicating that these markers were associated. Although lenvatinib may suppress FGF19‐mediated signaling, it may not suppress signaling mediated by ANG2 and HGF. Therefore, in patients with high levels of both FGF19 and ANG2, the response to lenvatinib may be inadequate. Further analysis is required.

To the best of our knowledge, the biomarkers for the treatment response to lenvatinib have not been elucidated. Numerous studies have analyzed the biomarkers of treatment response to sorafenib in patients with unresectable HCC. Several candidate biomarkers including HGF, VEGF, ANG2, and AFP have been reported; however, none of the validated biomarkers have been established in practice.^11^ The biomarker study on treatment response to sorafenib with the largest cohort of patients with unresectable HCC could not identify any biomarkers.^12^ As in previous studies, no significant predictors of treatment response to sorafenib were identified in the present cohort of patients with unresectable HCC. Therefore, the prediction of treatment response to sorafenib may be particularly challenging. However, the present study indicates that sorafenib may be suitable for patients with anticipated poor responses to lenvatinib owing to high FGF19 and ANG2 levels as the response is not affected by high FGF19 and ANG2 levels. Lenvatinib and sorafenib may be combined with immune checkpoint inhibitors for treating unresectable HCC in the near future.^29^ These findings may contribute to the selection of combination therapy in these cases.

Quite recently, Chuma et al., similar to our report, reported that serum ANG2 and FGF19 might be involved in treatment response of lenvatinib for patients with advanced HCC. However, in the report, they showed that, in patients with favorable responses, ANG2 level was higher at baseline but showed significant decrease along with significantly increased FGF19 levels during the treatment.^30^ The precise reason for this discrepancy in our results remains unclarified; however, there are several hypotheses. In this study, we included patients with Child‐Pugh grade B, while Chuma's study did not. We and other groups reported that not only the tumor itself^31^ but also advanced liver fibrosis could cause serum ANG2 elevation.^32^, ^33^ Thus, the difference in liver fibrosis and HCC condition might affect the results. Therefore, further analysis is required.

This study has several limitations. First, it was a retrospective single‐center study and included a limited sample size. Second, several baseline clinical factors, including age, platelet count, and presence of extrahepatic metastases, differed significantly between the sorafenib and lenvatinib groups; this must be considered when interpreting the results. However, this study is the *first* to demonstrate that baseline biomarkers may aid the selection of suitable systemic therapy for patients with unresectable HCC. This hypothesis requires validation in future prospective multicenter studies with large sample sizes.

In conclusion, the combination of baseline serum ANG2 and FGF19 levels may predict treatment response to lenvatinib in patients with unresectable HCC. An OR was achieved in all patients with low baseline levels of ANG2 and FGF19 treated with lenvatinib (100%, 9/9). Conversely, those with high baseline levels of these markers demonstrated a low OR rate (13%; 1/9). However, in patients with high baseline ANG2 and FGF19 levels, the response to sorafenib was not low (40% 2/5). These findings show, for the first time, that baseline biomarker levels may determine suitability for systemic therapy in patients with unresectable HCC. Further larger prospective studies are needed to validate our findings.

## Supporting information


**Table S1** Baseline patient characteristics
**Table S2** Comparison between HCC patients with or without high baseline ANG2 and FGF19 levels

**Figure S1** Study flow

**Figure S2** Treatment response to lenvatinib according to ANG2 (A) and FGF19 (B) cut‐off values. Baseline ANG2 and FGF19 levels exceeding or less than 3108 and 194 pg./mL were considered high and low, respectively

**Figure S3** Treatment response to sorafenib according to baseline ANG2 and FGF19 levels. Treatment responses were similar irrespective of baseline ANG2 and FGF19 levels
