# Supplementary material for: Baseline angiopoietin‐2 and FGF19 levels predict treatment response in patients receiving multikinase inhibitors for hepatocellular carcinoma
Source: JGH Open. 2020 Apr 11;4(5):880–8. doi: 10.1002/jgh3.12339 (PMC7578287; doi:10.1002/jgh3.12339)
Supplement: Supplementary file 1 — Table S1 Baseline patient characteristics Table S2 Comparison between HCC patients with or without high baseline ANG2 and FGF19 levels [file JGH3-4-880-s001.docx]

**Supplementary Table 1 Baseline patient characteristics**

|  | **Lenvatinib (n=27)** | **Sorafenib (n=29)** | **p-Value** |
| --- | --- | --- | --- |
| **Baseline characteristics**  **Age－(yr)**  **Sex (Male/Female)**  **Etiology －no.(%)**  HBV  HCV  NBNC  **Vascular invasion－no.(%)**  **Extrahepatic extension－no.(%)**  **BCLC stage－no.(%)**  B  C  **Child-Pugh class－no.(%)**  A  B  **Biochemical analysis**  **Albumin－g/dL**  **Total bilirubin－mg/dL**  **Prothrombin time －%**  **Platelet －*10^4^/μL**  **Alpha-fetoprotein－ng/mL**  **AFP－L3%**  **PIVKA-II－mAU/mL** | 68 (54-83)  25/2  10 (37%)  6 (22%)  11 (41%)  7 (26%)  6 (22%)  10 (37%)  17 (63%)  21 (78%)  6 (22%)  3.6 (2.8-4.6)  0.7 (0.3-3.1)  89.7 (46.6-117.1)  16.0 (4.4-51.7)  32 (1.6-449909)  31 (0.5-99.5)  1251.5 (13-195319) | 63 (38-89)  26/3  17 (58%)  6 (21%)  6 (21%)  11 (38%)  16 (55%)  10 (34%)  19 (66%)  23 (79%)  6 (21%)  3.8 (2.8-4.5)  0.9 (0.1-2.1)  84 (44-124)  10.3 (4.5-31.6)  81 (2-193374)  17.8 (0-93.0)  478 (17-106354) | **0.029**  >0.999  0.149  0.576  **0.015**  0.787  >0.999  0.157  0.220  0.294  **0.041**  0.133  0.468  0.694 |

yr: years old, HBV: Hepatitis B virus, HCV: Hepatitis C virus, NBNC: non-B non-C, BCLC: Barcelona Clinic Liver Cancer, AFP: Alpha-fetoprotein, PIVKA-Ⅱ: protein induced by vitamin K absence or antagonist-II

**Supplementary Table 2**

**Comparison between HCC patients with or without high baseline ANG2 and FGF19 level**

|  | **ANG2 High / FGF19 High**  **(n=13)** | **Others (n=43)** | **p-Value** |
| --- | --- | --- | --- |
| **Baseline characteristics**  **Age－yr**  **Sex (Male/Female)**  **Etiology －no.(%)**  HBV  HCV  NBNC  **Vascular invasion－no.(%)**  **Extrahepatic extension－no.(%)**  **BCLC stage－no.(%)**  B  C  **Child-Pugh class－no.(%)**  A  B  **Biochemical analysis**  **Albumin－g/dL**  **Total bilirubin－mg/dL**  **Prothrombin time －%**  **Platelet －*10^4^/μL**  **Alpha-fetoprotein－ng/mL**  **AFP－L3%**  **PIVKA-II－mAU/mL**  **VEGF－pg/mL**  **HGF－pg/mL** | 67 (53-83)  11/2  4 (31%)  3 (23%)  6 (46%)  3 (23%)  5 (38%)  4 (31%)  9 (69%)  10 (77%)  3 (23%)  3.3 (2.8-4.0)  0.7 (0.1-3.1)  88.4 (46.6-107.6)  20.3 (4.4-51.7)  188.6 (6.4-449909)  42.6 (0.5-81)  2040 (21-47270)  454.0 (73.6-1356.7)  3890 (3020.6-5613.6) | 67 (38-89)  40/3  23 (53%)  9 (21%)  11 (26%)  15 (35%)  17 (40%)  16 (37%)  27 (63%)  34 (79%)  9 (21%)  3.8 (2.8-4.6)  0.7 (0.1-2.1)  86.8 (44-124)  13.4 (4.5-34.6)  22 (1.6-193374)  17.6 (0-99.5)  408.5 (13-195319)  356.8 (84.0-1104.5)  3077 (1023.8-6723.5) | 0.863  0.580  0.289  0.514  >0.999  0.751  >0.999  **0.027**  0.828  >0.999  0.096  0.153  0.144  0.409  0.611  **0.005** |

ANG2: Angiopoietin 2, FGF19: Fibroblast growth factor 19, yr: years old, HBV: Hepatitis B virus, HCV: Hepatitis C virus, NBNC: non-B non-C, BCLC: Barcelona Clinic Liver Cancer,

AFP: Alpha-fetoprotein, PIVKA-Ⅱ: protein induced by vitamin K absence or antagonist-II, VEGF: Vascular endothelial growth factor, HGF: Hepatocyte growth factor
