# Supplementary figures and images for: Baseline angiopoietin‐2 and FGF19 levels predict treatment response in patients receiving multikinase inhibitors for hepatocellular carcinoma
Source: JGH Open. 2020 Apr 11;4(5):880–8. doi: 10.1002/jgh3.12339 (PMC7578287; doi:10.1002/jgh3.12339)

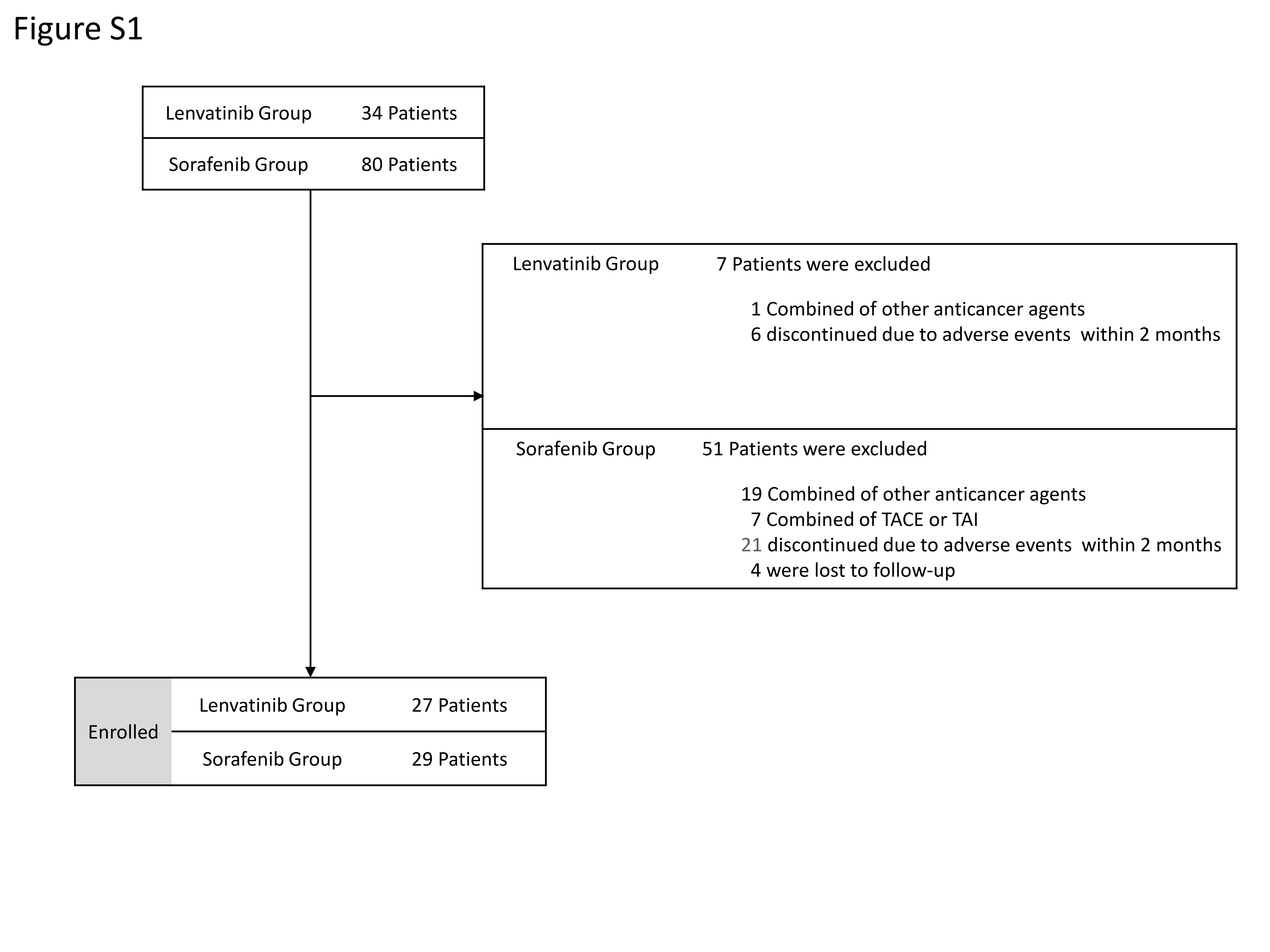

Supplement: Supplementary file 2 — Figure S1 Study flow [file JGH3-4-880-s002.tif]

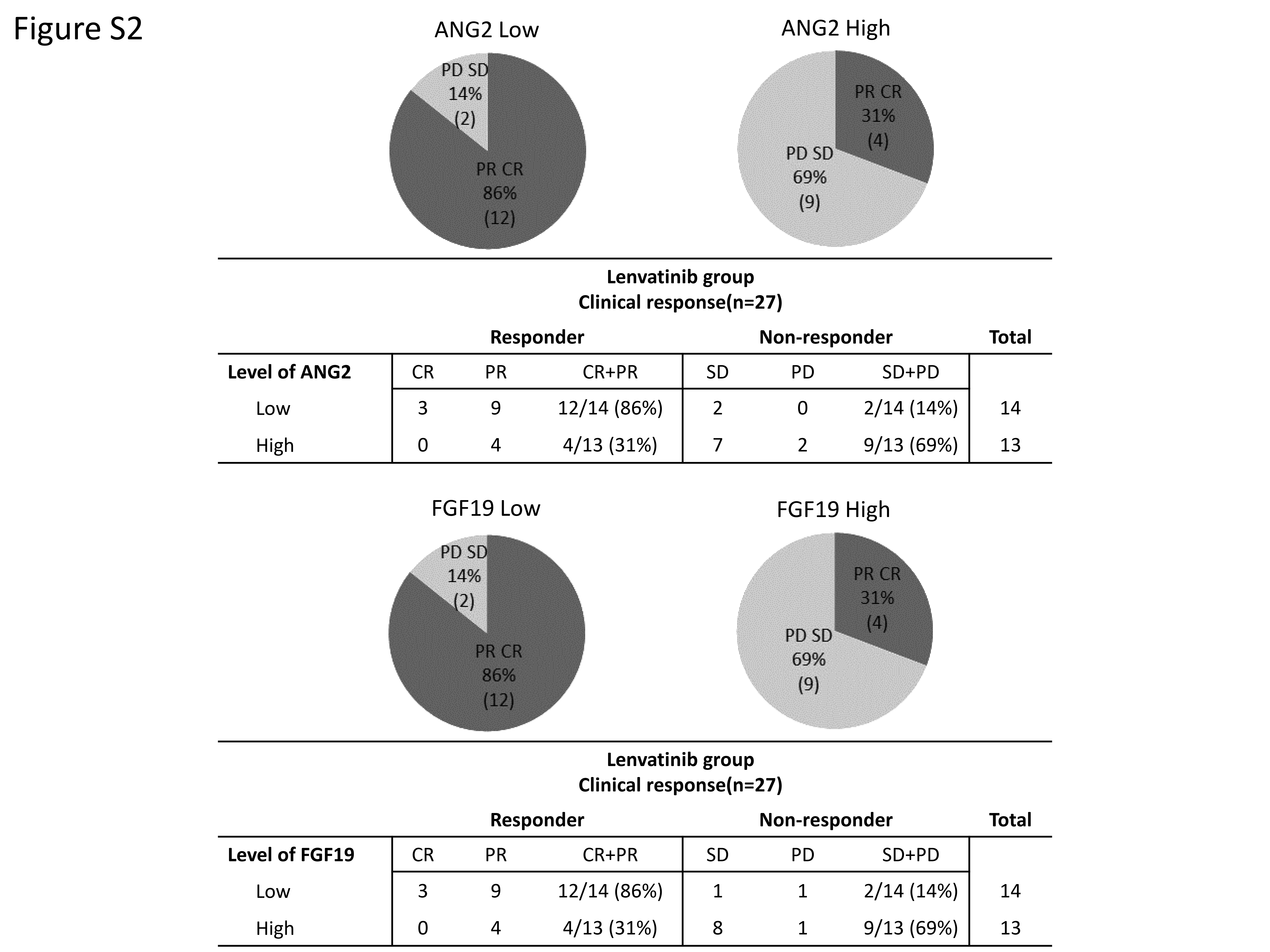

Supplement: Supplementary file 3 — Figure S2 Treatment response to lenvatinib according to ANG2 (A) and FGF19 (B) cut‐off values. Baseline ANG2 and FGF19 levels exceeding or less than 3108 and 194 pg./mL were considered high and low, respectively [file JGH3-4-880-s003.tif]

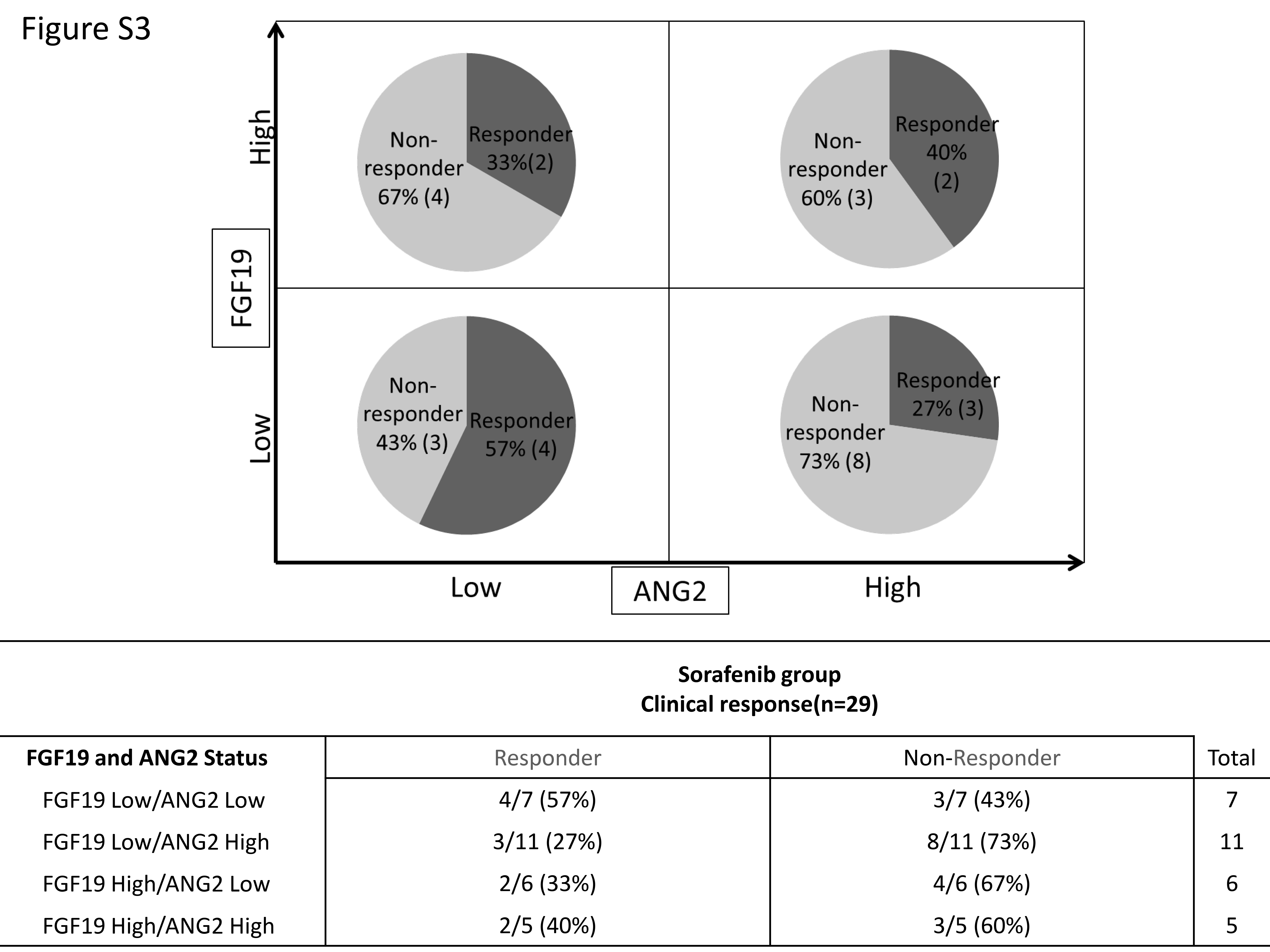

Supplement: Supplementary file 4 — Figure S3 Treatment response to sorafenib according to baseline ANG2 and FGF19 levels. Treatment responses were similar irrespective of baseline ANG2 and FGF19 levels [file JGH3-4-880-s004.tif]
